# Spinal afferent neurons projecting to the rat lung and pleura express acid sensitive channels

**DOI:** 10.1186/1465-9921-7-96

**Published:** 2006-07-01

**Authors:** Michael Groth, Tanja Helbig, Veronika Grau, Wolfgang Kummer, Rainer V Haberberger

**Affiliations:** 1 Institute for Anatomy and Cell Biology, University of Giessen Lung Center, Justus-Liebig-University, Giessen, Germany; 2 Department of General and Thoracic Surgery, Laboratory of Experimental Surgery, University of Giessen Lung Center, Justus-Liebig-University, Giessen, Germany

## Abstract

**Background:**

The acid sensitive ion channels TRPV1 (transient receptor potential vanilloid receptor-1) and ASIC3 (acid sensing ion channel-3) respond to tissue acidification in the range that occurs during painful conditions such as inflammation and ischemia. Here, we investigated to which extent they are expressed by rat dorsal root ganglion neurons projecting to lung and pleura, respectively.

**Methods:**

The tracer DiI was either injected into the left lung or applied to the costal pleura. Retrogradely labelled dorsal root ganglion neurons were subjected to triple-labelling immunohistochemistry using antisera against TRPV1, ASIC3 and neurofilament 68 (marker for myelinated neurons), and their soma diameter was measured.

**Results:**

Whereas 22% of pulmonary spinal afferents contained neither channel-immunoreactivity, at least one is expressed by 97% of pleural afferents. TRPV1^+^/ASIC3^- ^neurons with probably slow conduction velocity (small soma, neurofilament 68-negative) were significantly more frequent among pleural (35%) than pulmonary afferents (20%). TRPV1^+^/ASIC3^+ ^neurons amounted to 14 and 10% respectively. TRPV1^-^/ASIC3^+ ^neurons made up between 44% (lung) and 48% (pleura) of neurons, and half of them presumably conducted in the A-fibre range (larger soma, neurofilament 68-positive).

**Conclusion:**

Rat pleural and pulmonary spinal afferents express at least two different acid-sensitive channels that make them suitable to monitor tissue acidification. Patterns of co-expression and structural markers define neuronal subgroups that can be inferred to subserve different functions and may initiate specific reflex responses. The higher prevalence of TRPV1^+^/ASIC3^- ^neurons among pleural afferents probably reflects the high sensitivity of the parietal pleura to painful stimuli.

## Background

The lower respiratory tract receives primary afferent fibres both from vagal sensory (nodose and jugular) and dorsal root ganglia (DRG) that transmit information to the brainstem and spinal cord, respectively [1-5]. To date, the vagal airway afferents have received particular interest. Functionally, they can be grouped in rapidly adapting mechanoreceptors (RARs, also called "irritant receptors"), slowly adapting mechanoreceptors (SARs), and C-fibre afferents [1,6]. At least in the guinea-pig, the functionally diverse classes are segregated between the two vagal sensory ganglia, with SARs specifically located in the upper, jugular ganglion, and RARs specifically located in the lower, nodose ganglion [7]. Much less is known about afferents of the lower respiratory tract that have their nerve cell bodies in DRG. Neuronal tracing studies performed in rat, guinea-pig and mouse located neurons in cervical and upper thoracic DRG that send an axon to the lung [2-5], electrophysiological recordings identified pulmonary afferent pathways that traverse the sympathetic chain to enter the spinal cord via spinal nerves [8-10], and reflexes originating from the lower trachea and bronchi are not entirely abolished by vagotomy in cats and dogs [11-13]. Immunohistochemical studies on retrogradely labelled neurons indicate that pulmonary DRG neurons do not constitute a homogenous population [2,3,5]. Based on immunohistochemical investigation of DRG neurons retrogradely labelled from the mouse right bronchus, it has been concluded that the DRG pathway may have effects on the magnitude of neurogenic inflammation in airway diseases such as asthma [5]. With respect to the innervation of the pleura, even less is known. One study provided recordings from an in vitro preparation of rabbit phrenic nerve and mediastinal pleura and demonstrated multimodal mechano- and chemosensitive afferent units that respond to potentially tissue damaging stimuli [14]. A very recent study reports mechanosensitive, chemosensitive and multimodal properties of parietal pleura afferents travelling in the intercostal nerves of the rabbit [15]. On this background we set out to investigate the presence of acid-sensitive channels in rat DRG neurons projecting to the lung and pleura, since tissue acidification occurs during tissue damage and inflammation, and contributes to neuronal excitation under such conditions [16]. To this end, the neuronal tracer DiI (1,1'-dioctadecyl-3,3,3',3'-tetramethylindocarbocyanine) was injected either into the left lung or into the pleural cavity, and retrogradely labelled DRG neurons were immunohistochemically triple-labelled for the vanniloid receptor TRPV1 (transient receptor potential vanniloid receptor-1), the acid sensing ion channel-3 (ASIC3), and neurofilament 68 kDa (NF68), and their soma size was measured. Both, TRPV1 and ASIC3, are expressed by subsets of primary afferent neurons and are the major acid-sensitive ion channels of sensory neurons know so far [17-19]. TRPV1 is a proton-sensitive channel whose activity is modulated by heat and by the pungent ingredient of chilli peppers, capsaicin [20,21]. Targeted disruption of the TRPV1 gene in mice results in loss of calcitonin gene-related peptide release in response to acidified (pH 5.2–5.7) synthetic interstitial fluid in the murine heart [22]. In human airways, TRPV1 has been considered to contribute to an enhanced cough reflex and the cough response in chronic persistent cough of diverse causes [23]. ASIC3 is a proton-sensitive channel which has been implicated in nociception [24-26] but also in mechanoreception [27]. It is activated at higher pH (6.0) than TRPV1, and its targeted disruption does not influence acid-induced neuropeptide release [22] but abolished acid-induced hypersensitivity of muscle afferents [25]. In addition to their immunoreactivity to TRPV1 and ASIC3, the size of retrogradely labelled neuronal somata and their immunoreactivity for NF68 were recorded since these parameters allow conclusions on the conduction velocity of sensory neurons [28-31].

## Methods

### Animals and tracer application

This study was performed on 10 female Wistar rats (200–260 g body weight; Harlan Winkelmann, Borchen, Germany). The experiments were conducted in accordance with the European Communities Council Directive of 24 November 1986 (86/609/EEC). For tracing of pleural afferents, animals were initially anaesthetized by isofluran inhalation (Forene, Abbott, Wiesbaden, Germany), and then received an intramuscular injection of atropine (0.25 mg/kg body weight; Braun, Melsungen, Germany), and intraperitoneal injection of ketamine hydrochloride (90 mg/kg; Ketavet, Pharmacia and Upjohn, Erlangen, Germany) and medetomidin hydrochloride (0.1 mg/kg; Dormitor, Pfizer, Karlsruhe, Germany) and were ventilated (Harvard Rodent Ventilator G836, Harvard Apparatus, South Natick, MA, USA) via an endotracheal tubus at a frequency of 97/min with a stroke volume of 1 ml/100 g and a positive end-expiratory pressure of 5 cm H_2_O. Thoracotomy was performed at the level of the 4^th ^to 6^th ^intercostal space. Only a single intercostal space was opened per individual animal. The ventilation was shortly arrested to allow collapse of the lung, and tracer (1,1'-dioctadecyl-3,3,3',3'-tetramethylindocarbocyanine perchlorhydrate [DiI], Molecular Probes Europe, Leiden, NL; 0.25 ‰ in N, N'-dimethylformamide, Fluka, Buchs, Switzerland) was applied each caudally (2 μl) and cranially (2 μl) onto the costal pleural surface with a microsyringe (Hamilton, Bonaduz, Switzerland). Ventilation with positive end-expiratory pressure was continued, muscles and skin layers were sutured, and animals received a subcutaneous injection of atipamezol hydrochloride (0.5 mg/kg; Antisedan, Pfizer, Karlsruhe, Germany) to terminate anaesthesia. Until full recovery from anaesthesia, animals were placed on a warming pad. Animals were sacrificed by inhalation of an overdose sevoflurane (Sevorane; Abbott, Wiesbaden, Germany) 6 days after tracer application.

For tracing of pulmonary afferents, animals received intramuscular injection of atropine (0.05 mg/kg), xylazine hydrochloride (12 mg/kg; Rompun, Bayer, Leverkusen, Germany) and ketamine hydrochloride (80 mg/kg; Ketamin, Inresa, Freiburg, Germany). The trachea was exposed by a midline cervical incision and opened between two cartilage rings with a 26 G cannula. A 10 μl Hamilton syringe loaded with 5 μl of tracer was inserted through this slit into the tracheal lumen, preceded into the left stem bronchus, and then pushed into the lung parenchyma to inject the tracer. The wound was closed, and the animals were allowed to recover under controlled temperature and sacrificed at the 6^th ^postoperative day.

In a control experiment, an animal was anaesthetized as described for pulmonary tracing and the operative approach was identical to that for pleural tracing except that the intercostal muscle was left intact and the pleural cavity was not opened. In this experiment, 2 μl of tracer were injected into the 6^th ^intercostal muscle. The wound was closed, and the animal was allowed to recover and sacrificed at the 6^th ^postoperative day.

### Tissue processing and immunohistochemistry

Animals were killed by inhalation of an overdose of sevorane, and perfused via the left ventricle first with heparin-containing rinsing solution [32], and then with fixative (4% phosphate-buffered parafomaldehyde, pH 7.4 in 3 cases of pleura tracing, in 1 case of lung tracing, and in the intercostal muscle control experiment; Zamboni's fixans = 2% paraformaldehyde/15% saturated picric acid in 0.1 M phosphate buffer, pH 7.4, in 2 cases of pleura and 3 cases of lung tracing). Animals were kept for 1 h at 6°C, and then the following specimens were dissected: all thoracic viscera *en bloc*, DRG bilaterally at segmental levels lung: C4-Th1, sensory vagal (nodose-jugular) ganglia bilaterally, spinal cord at segmental levels C3-Th7, and, in case of pleura and intercostal muscle tracing experiments, the sites of tracer application. Specimens were rinsed repeatedly in 0.1 M phosphate buffer, and then placed for 24 h in this buffer containing 18% sucrose. Thereafter, specimens were mounted on filter paper in OCT compound (Tissue Tek, Sakura, Zoeterwoude, NL), frozen in liquid nitrogen, and stored at -80°C until sectioning.

Serial 10 μm-thick sections were cut on a cryotome (Jung Frigocut 2800E, Leica, Bensheim, Germany) and mounted on SuperFrost Plus slides (R. Langenbrinck, Emmendingen, Germany). A first screening for retrogradely labelled, DiI containing neurons was done with an epifluorescence microscope (530–560 nm excitation filter, 573–648 nm barrier filter; Axioplan 2, Zeiss, Jena, Gemany) before immunolabelling, and all DiI-containing neurons with visible nucleus were documented with a digital camera (Axiocam, Zeiss,) and their soma size was determined with Axio Vision Software (Zeiss). Only sections from left DRG at levels Th3-6 in case of pleura tracing and levels Th1-2 in case of lung tracing, respectively, which contained retrogradely labelled neurons were subjected to further immunolabelling. Non-specific protein binding sites were blocked with 0.1% bovine serum albumin, 10% normal porcine serum, 0.2% Tween 20 in 0.005 M phosphate buffer with 4.48 g/l NaCl (PBS). Sections were then incubated overnight at 4°C with a mixture of anti-ASIC3 from guinea pig (1:200; Chemicon, Hofheim, Germany), anti-TRPV1 from rabbit (1:2,000; Chemicon), and mouse monoclonal anti-NF68 (clone NR4, 27.5 μg/ml; Sigma, Steinheim, Germany). Following buffer washes, triple-labelling was achieved by simultaneous incubation (1 h, room temperature) with species-specific antisera raised in donkey against guinea pig Ig (conjugated to fluorescein isothiocyanate = FITC; 1:100), rabbit Ig (conjugated to Cy5; 1:50) and mouse Ig (conjugated to 7-amino-4-methylcoumarin-3-acetate = AMCA; 1:200) (all from Dianova, Hamburg, Germany). Routine techniques to assess species-specificity of secondary antisera [33] were applied to DRG sections without retrogradely labelled neurons. Washed sections were coverslipped in carbonate-buffered glycerol (pH 8.6), and retrogradely labelled neurons, which often lost much of the DiI fluorescence in the course of immunohistochemical processing, were re-identified by aid of the images taken before immunohistochemistry and assessed for their pattern of immunoreactivity. Filter combinations for epifluorescence microscopy were 340–380 nm excitation and 435–685 nm barrier filter for AMCA, 460–500 nm excitation and 512–542 nm barrier filter for FITC, and 590–650 nm excitation and 663–738 nm barrier filter for Cy5.

### Statistical methods

Relative frequencies of neurochemical characteristics of neurons retrogradely labelled from pleura and lung, respectively, were compared by the Chi^2^-test. Size distributions of neurochemically defined classes of neurons were compared by the Kolmogoroff-Smirnoff-test. Throughout, p < 0.05 was set as level for significance.

## Results

### Pleura tracing

#### Tracer distribution

**Figure 1:**
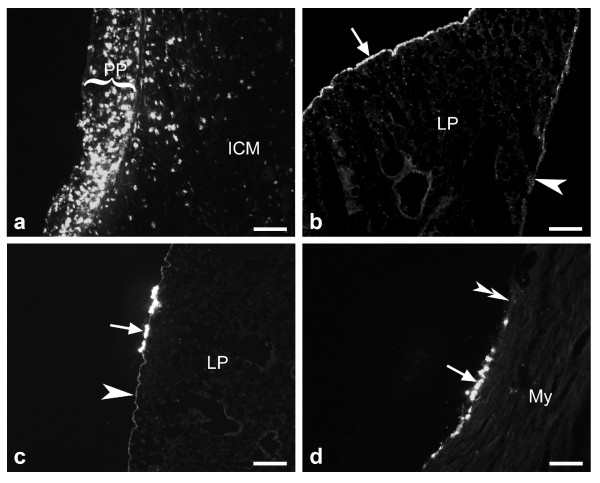
Tracer (DiI) distribution in thoracic tissues 6 days after application into the left pleural cavity. A) Close to the injection site, the parietal pleura (PP) is thickened and still contains an accumulation of DiI positive cells. ICM = intercostal muscle. B) The visceral pleura is more intensely labelled at the dorso-costal lung surface (*arrow*) than at its mediastinal surface (*arrowhead*). C, D) Solitary spots of DiI (arrows) are also seen at the visceral pleura of the contralateral lung (C) and the pericardium (D) while most contralateral pleural (C, arrowhead) and pericardial stretches (D, doubled arrowhead) have not accumulated tracer. LP = lung parenchyma, My = myocardium. Bars represent 100 μm in *A *and *D*, and 200 μm in *B *and *C*.

As described in the methods section, tracer application onto the costal pleura of the left pleural cavity was performed with collapsed lung, and artificial respiration with positive end-expiratory pressure was continued immediately thereafter. At the time of sacrifice, a DiI spot was still macroscopically visible at the parietal pleura at the application site (segmental level Th4: n = 1, Th5: n = 2, Th6: n = 2). In this region, fluorescence microscopy revealed pleural thickening and intense DiI accumulation (Fig. 1A). DiI was also observed at the visceral pleural lining of the dorso-costal surface of the left lung whereas the mediastinal visceral pleura was less labelled (Fig. 1B). Screening of the contralateral lung (Fig. 1C) and of the pericardium (Fig. 1D) revealed single small spots of DiI also at these locations. In the control experiment where DiI was injected into the left 6^th ^intercostal muscle, tracer was restricted to the muscle and did not appear at the pulmonary surface or pericardium.

**Figure 2:**
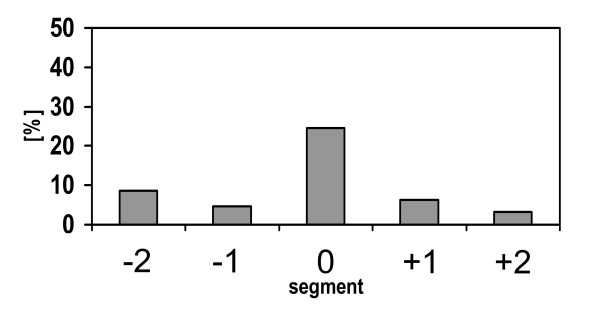
Distribution of retrogradely labelled DRG neurons after tracer injection into the pleural cavity. Segmental level "0" depicts that level at which the thorax was opened for tracer application (Th4: n = 1; Th5: n = 2, Th6: n = 2), level "-1" designates the DRG located immediately cranial, level "1" designates the DRG located immediately caudal to this level. Relative frequencies are shown, total number of neurons = 160, taken from 4 animals. Data from animal #4 are excluded since in this experiment DRG Th5 (left side) was lost during tissue processing (cf. Table 1).

After application of DiI onto the costal pleura, retrogradely labelled neurons were found in DRG at different segmental levels (Tab. 1) and in the jugular-nodose complex (Tab. 2) at both sides. The number of ipsilaterally located neurons amounted twice that of contralateral neurons (relative frequencies of ipsilateral neurons: 64% in DRG, 67% in the jugular-nodose complex). Ipsilaterally, about 25% of retrogradely labelled DRG neurons were located at the segmental level of application, and roughly the same number was distributed over the 4 adjacent (2 cranial and 2 caudal) DRG (Fig. 2). Smaller, additional peaks in the numbers of DiI-labelled neurons were found at levels C4-C6 and Th1-Th2 (Tab. 1). Labelled neurons were not observed in the spinal cord from C3 to Th7.

**Table 1:** Segmental distribution of retrogradely labelled DRG neurons after DiI injection into the left pleural cavity

**Left**	**Rat 1**		**Rat 2**		**Rat 3**		**Rat 4**		**Rat 5**	
	
	**n**	**%**	**n**	**%**	**n**	**%**	**n**	**%**	**n**	**%**
**C2**	3	*2.8*	0	*0*	0	*0*	X	*X*	X	*X*
**C3**	0	*0*	0	*0*	0	*0*	1	*3.2*	0	*0*
**C4**	3	*2.8*	7	*7.6*	4	*4.7*	0	*0*	0	*0*
**C5**	17	*15.7*	5	*5.4*	14	*16.3*	0	*0*	12	*23.5*
**C6**	0	*0*	3	*3.3*	4	*4.7*	6	*19.4*	0	*0*
**C7**	1	*0.9*	0	*0*	0	*0*	1	*3.2*	1	*2.0*
**C8**	0	*0*	5	*5.4*	0	*0*	0	*0*	3	*5.9*
**Th1**	12	*11.1*	18	*19.6*	5	*5.8*	0	*0*	0	*0*
**Th2**	13	*12.0*	4	*4.4*	2	*2.3*	1	*3.2*	6	*11.8*
**Th3**	7	*6.5*	8	*8.7*	1	*1.2*	4	*12.9*	3	*5.9*
**Th4**	24	*22.2*	5	*5.4*	5	*5.8*	3	*9.7*	3	*5.9*
**Th5**	8	*7.4*	26	*28.3*	2	*2.3*	X	*X*	2	*3.9*
**Th6**	2	*1.9*	0	*0*	22	*25.6*	12	*38.7*	11	*21.6*
**Th7**	5	*4.6*	3	*3.3*	6	*7.0*	2	*6.5*	7	*13.7*
**Th8**	4	*3.7*	2	*2.2*	4	*4.7*	0	*0*	2	*3.9*
**Th9**	0	*0*	6	*6.5*	5	*5.8*	0	*0*	0	*0*
**Th10**	0	*0*	0	*0*	4	*4.7*	1	*3.2*	1	*2.0*
**Th11**	4	*3.7*	0	*0*	4	*4.7*	0	*0*	0	*0*
**Th12**	5	*4.6*	0	*0*	4	*4.7*	0	*0*	0	*0*
**L1**	0	*0*	0	*0*	X	*X*	0	*0*	0	*0*

Σ	108	*100*	92	*100*	86	*100*	31	*100*	51	*100*

										

**Right**	**Rat 1**		**Rat 2**		**Rat 3**		**Rat 4**		**Rat 5**	
	
	**n**	**%**	**n**	**%**	**n**	**%**	**n**	**%**	**n**	**%**

**C2**	3	*3.7*	0	*0*	0	*0*	0	*0*	1	*5.9*
**C3**	0	*0*	0	*0*	2	*3.5*	2	*6.5*	0	*0*
**C4**	3	*3.7*	0	*0*	2	*3.5*	0	*0*	2	*11.8*
**C5**	7	*8.5*	1	*4.4*	7	*12.1*	0	*0*	1	*5.9*
**C6**	7	*8.5*	0	*0*	3	*5.2*	3	*9.7*	0	*0*
**C7**	1	*1.2*	0	*0*	0	*0*	0	*0*	0	*0*
**C8**	0	*0*	1	*4.4*	0	*0*	0	*0*	0	*0*
**Th1**	18	*22.0*	5	*21.7*	8	*13.8*	0	*0*	0	*0*
**Th2**	8	*9.8*	4	*17.4*	11	*19.0*	0	*0*	0	*0*
**Th3**	3	*3.7*	2	*8.7*	14	*24.1*	5	*16.1*	0	*0*
**Th4**	25	*30.5*	8	*34.8*	1	*1.7*	1	*3.2*	3	*17.7*
**Th5**	2	*2.4*	0	*0*	0	*0*	10	*32.3*	8	*47.1*
**Th6**	0	*0*	0	*0*	3	*5.2*	3	*9.7*	2	*11.8*
**Th7**	0	*0*	1	*4.4*	1	*1.7*	6	*19.4*	0	*0*
**Th8**	1	*1.2*	1	*4.4*	0	*0*	1	*3.2*	0	*0*
**Th9**	1	*1.2*	0	*0*	1	*1.7*	0	*0*	0	*0*
**Th10**	0	*0*	0	*0*	3	*5.2*	0	*0*	0	*0*
**Th11**	2	*2.4*	0	*0*	2	*3.5*	0	*0*	0	*0*
**Th12**	1	*1.2*	0	*0*	0	*0*	0	*0*	0	*0*
**L1**	0	*0*	0	*0*	0	*0*	0	*0*	0	*0*

Σ	82	*100*	23	*100*	58	*100*	31	*100*	17	*100*

**Table 2:** Number and distribution of retrogradely labelled sensory vagal neurons after DiI injection into the left pleural cavity

	**Rat 1**		**Rat 2**		**Rat 3**		**Rat 4**		**Rat 5**		Σ	
	
	**n**	**%**	**n**	**%**	**n**	**%**	**n**	**%**	**n**	**%**	**n**	**%**
**left**	13	*65*	12	*66.7*	7	*70*	12	*57.1*	23	*74.2*	67	*67*
**right**	7	*35*	6	*33.3*	3	*30*	9	*42.9*	8	*25.8*	33	*33*

Σ	20	*100*	18	*100*	10	*100*	21	*100*	31	*100*	100	*100*

In contrast, DiI injection into the 6^th ^intercostal muscle resulted in purely ipsilateral labelling of DRG neurons with restriction to the segment of injection (83%) and its immediate cranial and caudal neighbours (16%) (Tab. 3), and intensely labelled motoneurons were observed in the spinal ventral column from Th5 to Th8.

**Table 3:** Segmental distribution of retrogradely labelled DRG neurons after DiI injection into the left 6^th ^intercostal muscle.

	**Th1**		**Th2**		**Th3**		**Th4**		**Th5**		**Th6**		**Th7**		**Th8**		**Th9**		**Th10**		**Th11**		**Th12**		Σ	
	
	**n**	**%**	**n**	**%**	**n**	**%**	**n**	**%**	**n**	**%**	**n**	**%**	**n**	**%**	**n**	**%**	**n**	**%**	**n**	**%**	**n**	**%**	**n**	**%**	**n**	**%**
**left**	0	0	1	0	0	0	1	0	14	5	231	83	30	11	0	0	1	0	0	0	0	0	0	0	278	100
**right**	0	0	0	0	0	0	0	0	0	0	0	0	0	0	0	0	0	0	0	0	0	0	0	0	0	0

Σ	0	0	1	0	0	0	1	0	14	5	231	83	30	11	0	0	1	0	0	0	0	0	0	0	278	100

### Size and neurochemical characteristics of retrogradely labelled DRG neurons

**Figure 3:**
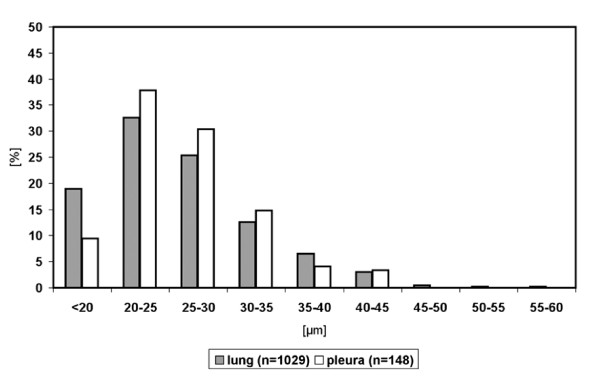
Size distribution of DRG neurons retrogradely labelled from lung (n = 1,030 neurons located in DRG Th1-Th2, left side) and pleura (n = 148 neurons located in DRG Th3-Th6, left side).

Size measurements of neurons retrogradely labelled after tracer application onto the costal pleura were performed on 148 neurons in ipsilateral DRG at segmental levels Th3-Th6. Two thirds of retrogradely labelled neurons were in the size range between 20 and 30 μm (20–25 μm: 38%, 25–30 μm: 30%), and only 7% were larger than 35 μm. Retrogradely labelled neurons larger than 45 μm were not observed (Fig. 3).

**Figure 4:**
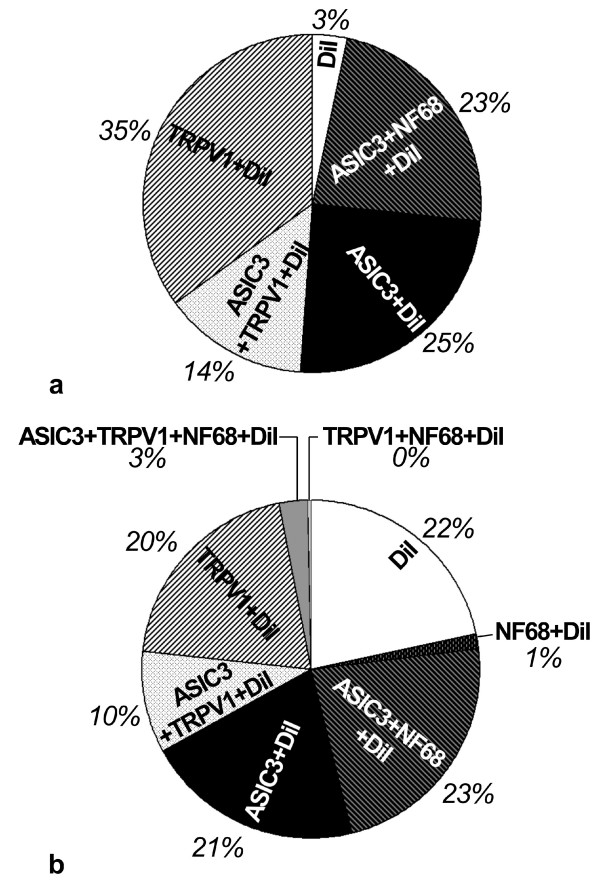
Relative frequencies of neurochemically characterized subpopulations of DRG neurons retrogradely labelled from pleura (A; n = 94) and lung (B; n = 761).

**Figure 5:**
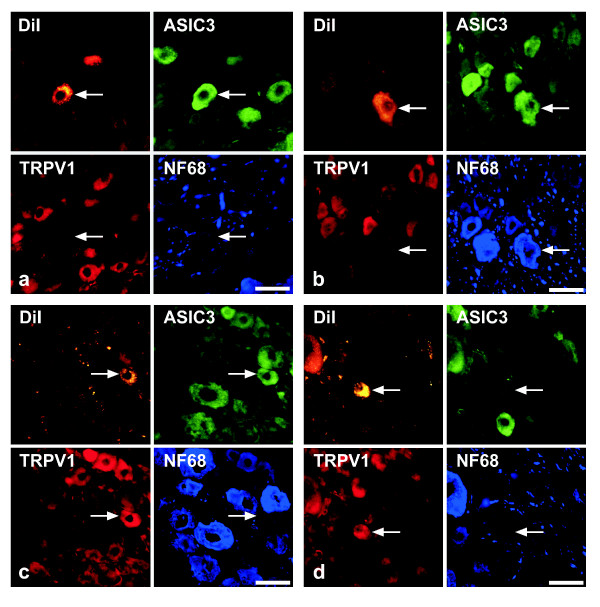
Quadruple-labelling (accumulation of fluorescent tracer plus triple-labelling immunohistochemistry) of DRG neurons after DiI injection into the pleural cavity. The most frequent patterns (cf. also fig. 4) of immunolabelling of DiI-positive neurons (*arrow*) are A) ASIC3^+^/TRPV1^-^/NF68^-^, B) ASIC3^+^/TRPV1^-^/NF68^+^, C) ASIC3^+^/TRPV1^+^/NF68^-^, and D) ASIC3^-^/TRPV1^+^/NF68^+^. Bar represents 50 μm throughout.

Successful immunohistochemical triple-labelling could be obtained for 94/148 of the neurons. In the order of frequency, the following combinations of immunoreactivities to ASIC3, TRPV1, and NF68 in DiI-positive cells (neurons/perikarya) were found (cf. Fig. 4): ASIC3^-^/TRPV1^+^/NF68^- ^(Fig. 5D), ASIC3^+^/TRPV1^-^/NF68^- ^(Fig. 5A), ASIC3^+^/TRPV1^-^/NF68^+ ^(Fig. 5B), ASIC3^+^/TRPV1^+^/NF68^- ^(Fig. 5C), and triple-negative. Thus, NF68-immunoreactivity was observed only in combination with ASIC3-immunoreactivity, and only 3% of neurons (triple-negative) contained neither ASIC3- nor TRPV1-immunoreactivity.

**Figure 6:**
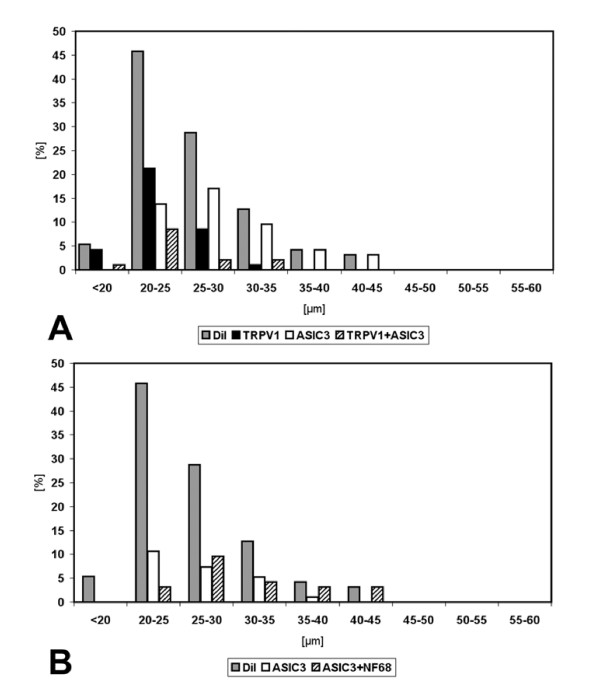
Size distributions of neurochemically characterized subpopulations of DRG neurons retrogradely labelled from pleura. In both panels, shaded columns designated "DiI" provide the size distribution of all retrogradely labelled neurons from which immunohistochemical data could be successfully collected (n = 94), regardless of their pattern of immunoreactivity. In *A*, neurons with different expression patterns of acid sensitive channels are compared, showing that neurons with TRPV1, either alone or in combination with ASIC3, have a peak in a smaller size group (20–25 μm) than those with ASIC3 alone. The latter are not subdivided in this panel according to their NF68-immunoreactivity. This is done in *B*, demonstrating larger size of NF68-positive compared to NF68-negative ASIC3-immunoreactive neurons.

Both populations of TRPV1-immunoreactive neurons, i.e. those with and those without additional ASIC3-immunoreactivity, had a peak frequency in the size range of 20–25 μm and did not exceed 35 μm in diameter (Fig. 6A). ASIC3^+^/TRPV1^- ^neurons without TRPV1-immunoreactivity, on the other hand, had a broader size distribution reaching up to 45 μm with a peak at 25–30 μm (Fig. 6A). Subdivision of ASIC3^+ ^neurons into those with and those without NF68-immunoreactivity revealed that NF68^+ ^neurons ranged from 20–45 μm with a peak at 25–30 μm, whereas NF68^- ^neurons ranged from below 20 to 40 μm with a peak at 20–25 μm (Fig. 6B). The size distribution of ASIC3^+^/NF68^+ ^neurons differed significantly from those of ASIC3^-^/TRPV1^+ ^(p < 0.01; Kolmogoroff-Smirnoff-test) and ASIC3^+^/TRPV1^+ ^neurons (p < 0.05; Kolmogoroff-Smirnoff-test), whereas no significant differences were observed for other couples.

### Lung tracing

#### Tracer distribution

**Figure 7:**
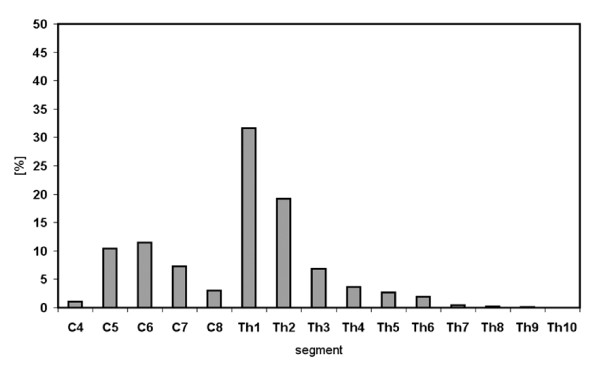
Distribution of retrogradely labelled DRG neurons of the left side after tracer injection into the left lung.

The cannula of a Hamilton syringe was inserted into the trachea, and preceded into the left lung parenchyma where the tracer DiI was delivered. At the time of sacrifice, DiI was not macroscopically visible in the lung. Fluorescence microscopical investigation revealed the expected tracer distribution in the left lung and, to a very minor extent, also in airways of the right lung. Retrogradely labelled neurons were found in DRG at segmental levels C4-Th10 of both sides, with only 1% of neurons located caudally to Th6 (Tab. 4). A clear peak in frequency was observed at segmental level Th1/2, where 51% of all DiI positive neurons were observed on the left side (Fig. 7; Tab. 4). An additional minor peak was found at C6 (Fig. 7). Eighty percent of retrogradely labelled neurons were located ipsilaterally. Complete series of sections of sensory vagal ganglia were obtained for animals #2 and #3 only, and in these cases the number of retrogradely labelled vagal neurons amounted 60% of that found in total in DRG (Tab. 5). In each animal, very occasionally (<10 neurons per case) retrogradely labelled motoneurons were observed in the ventral column of the spinal cord at around segmental level C3.

**Table 4:** Segmental distribution of retrogradely labelled DRG neurons after DiI injection into the left lung.

**Left**	**Rat 1**		**Rat 2**		**Rat 3**		**Rat 4**		**mean**
	
	**n**	**%**	**n**	**%**	**n**	**%**	**n**	**%**	**%**
**C4**	0	*0.0*	4	*0.6*	12	*2.3*	5	*0.9*	*1.0*
**C5**	4	*1.5*	94	*13.7*	40	*7.5*	73	*13.5*	*9.1*
**C6**	31	*11.7*	102	*14.9*	66	*12.4*	33	*6.1*	*11.3*
**C7**	36	*13.6*	25	*3.6*	47	*8.8*	40	*7.4*	*8.4*
**C8**	14	*5.3*	18	*2.6*	21	*3.9*	8	*1.5*	*3.3*
**Th1**	32	*12.1*	305	*44.5*	167	*31.3*	136	*25.1*	*28.3*
**Th2**	109	*41.3*	90	*13.1*	103	*19.3*	87	*16.1*	*22.5*
**Th3**	19	*7.2*	19	*2.8*	48	*9.0*	52	*9.6*	*7.2*
**Th4**	14	*5.3*	16	*2.3*	15	*2.8*	28	*5.2*	*3.9*
**Th5**	0	*0.0*	8	*1.2*	8	*1.5*	39	*7.2*	*2.5*
**Th6**	5	*1.9*	3	*0.4*	2	*0.4*	30	*5.6*	*2.1*
**Th7**	0	*0.0*	2	*0.3*	2	*0.4*	5	*0.9*	*0.4*
**Th8**	0	*0.0*	0	*0.0*	0	*0.0*	4	*0.7*	*0.2*
**Th9**	0	*0.0*	0	*0.0*	1	*0.2*	1	*0.2*	*0.1*
**Th10**	0	*0.0*	0	*0.0*	1	*0.2*	0	*0.0*	*0.1*

Σ	264	*100*	686	*100*	533	*100*	541	*100*	*100*

									

**Right**	**Rat 1**		**Rat 2**		**Rat 3**		**Rat 4**		**mean**
	
	**n**	**%**	**n**	**%**	**n**	**%**	**n**	**%**	**%**

**C4**	2	*9.5*	20	*5.8*	X	*X*	2	*2.9*	*6.1*
**C5**	3	*14.3*	85	*24.5*	38	*19.6*	1	*1.5*	*13.4*
**C6**	0	*0.0*	44	*12.7*	31	*16.0*	0	*0.0*	*4.2*
**C7**	0	*0.0*	2	*0.6*	1	*0.5*	1	*1.5*	*0.7*
**C8**	1	*4.8*	9	*2.6*	1	*0.5*	1	*1.5*	*3.0*
**Th1**	4	*19.1*	101	*29.1*	43	*22.2*	10	*14.5*	*20.9*
**Th2**	11	*52.4*	78	*22.5*	64	*33.0*	15	*21.7*	*32.2*
**Th3**	0	*0.0*	5	*1.4*	X	*X*	4	*5.8*	*2.4*
**Th4**	0	*0.0*	1	*0.3*	12	*6.2*	8	*11.6*	*4.0*
**Th5**	0	*0.0*	1	*0.3*	2	*1.0*	9	*13.0*	*4.4*
**Th6**	0	*0.0*	0	*0.0*	1	*0.5*	15	*21.7*	*7.2*
**Th7**	0	*0.0*	0	*0.0*	0	*0.0*	1	*1.5*	*0.5*
**Th8**	0	*0.0*	1	*0.3*	1	*0.5*	1	*1.5*	*0.6*
**Th9**	0	*0.0*	0	*0.0*	0	*0.0*	1	*1.5*	*0.5*
**Th10**	0	*0.0*	0	*0.0*	0	*0.0*	0	*0.0*	*0.0*

Σ	21	*100*	347	*100*	194	*100*	69	*100*	*100*

**Table 5:** Number and distribution of retrogradely labelled sensory vagal neurons after DiI injection into the left lung.

	**Rat 1**		**Rat 2**		**Rat 3**		**Rat 4**		Σ	
	
	**n**	**%**	**n**	**%**	**n**	**%**	**n**	**%**	**n**	**%**
**left**	135	X	345	54.6	247	59.8	X	X	296	57.2
**right**	X	X	287	45.4	166	40.2	95	X	226.5	42.8

Σ	X	X	632	100	413	100	X	X		100,0

### Size and neurochemical characteristics of retrogradely labelled DRG neurons

Size measurements were performed on retrogradely labelled neurons in ipsilateral DRG at segmental levels C6 and C7, Th1 and Th2 (n = 1,030; Fig. 3), and Th4 and Th5. Size distributions were practically identical at these levels and, accordingly, no significant differences were disclosed by the Kolmogoroff-Smirnoff-test. At segmental level Th1/2 where the majority of labelled neurons was located, size range peaked between 20 and 25 μm in diameter (33%), and 58% of labelled neurons measured between 20 and 30 μm (Fig. 3). About 20% of neurons were smaller than 20 μm, and only 7/1,030 had a diameter greater than 45 μm.

**Figure 8:**
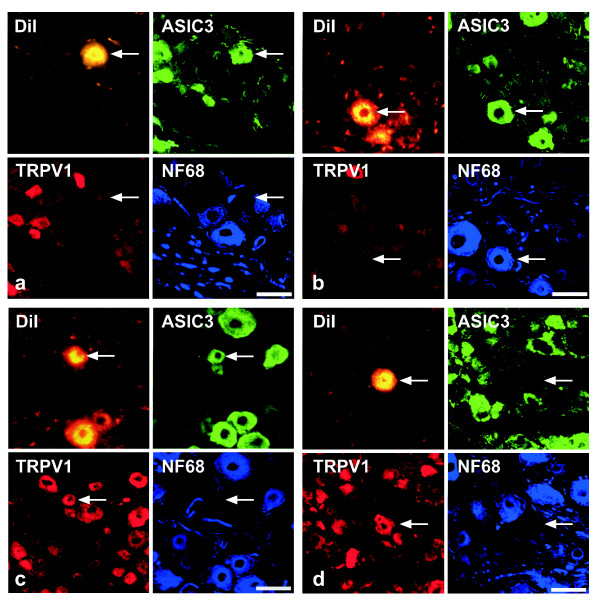
Quadruple-labelling (accumulation of fluorescent tracer plus triple-labelling immunohistochemistry) of DRG neurons after DiI injection into the pleural cavity, showing A) ASIC3^+^/TRPV1^-^/NF68^-^, B) ASIC3^+^/TRPV1^-^/NF68^+^, C) ASIC3^+^/TRPV1^+^/NF68^-^, and D) ASIC3^-^/TRPV1^+^/NF68^+ ^patterns of immunoreactivity. Bar represents 50 μm throughout.

Successful immunohistochemical triple-labelling could be obtained for 761 of the neurons. Those combinations of immunoreactivities to ASIC3, TRPV1 and NF68, that were found in DRG neurons retrogradely labelled from the pleural cavity, i.e. ASIC3^-^/TRPV1^+^/NF68^-^, ASIC3^+^/TRPV1^-^/NF68^-^, ASIC3^+^/TRPV1^-^/NF68^+^, ASIC3^+^/TRPV1^+^/NF68^-^, and triple-negative, were also observed in DiI positive neurons after lung injection (Fig. 8), and amounted to 95% of all retrogradely labelled neurons (Fig. 4). In addition, 21/761 (2.8%) DiI positive neurons were triple-labelled for ASIC3, TRPV1 and NF68, 11/761 (1.4%) for NF68 only, and 2/761 (0.3%) for TRPV1 plus NF68 (Fig. 8).

**Figure 9:**
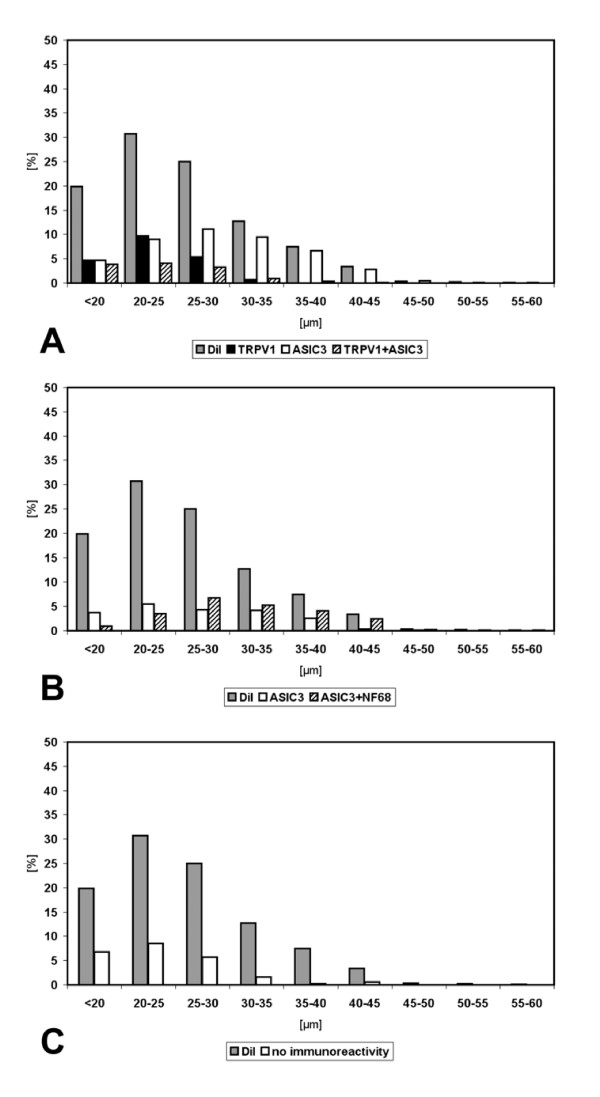
Size distributions of neurochemically characterized subpopulations of DRG neurons retrogradely labelled from the left lung. In all panels, shaded columns designated "DiI" provide the size distribution of all retrogradely labelled neurons, regardless of their pattern of immunoreactivity. In *A*, neurons with different expression patterns of acid sensitive channels are compared, showing that neurons with TRPV1, either alone or in combination with ASIC3, have a peak in a smaller size group (20–25 μm) than those with ASIC3 alone. The latter are not subdivided in this panel according to their NF68-immunoreactivity. This is done in *B*, demonstrating larger size of NF68-positive compared to NF68-negative ASIC3-immunoreactive neurons. C. Triple-immunonegative retrogradely labelled neurons were predominantly small to medium-sized.

TRPV1-immunoreactive neurons without additional ASIC3-immunoreactivity had a peak frequency in the size range of 20–25 μm, and one individual neuron exceeded 35 μm in diameter (Fig. 9A). TRPV1-immunoreactive neurons with additional ASIC3-immunoreactivity, however, occurred at similar frequencies in the size ranges below 20 μm, 20–25 μm, and 25–30 μm, respectively (Fig. 9A). ASIC3^+ ^neurons without TRPV1-immunoreactivity had a broader size distribution reaching up to 60 μm with a peak at 25–30 μm (Fig. 9A). Subdivision of ASIC3^+ ^neurons into those with and those without NF68-immunoreactivity revealed that NF68^+ ^neurons covered the whole size range up to 60 μm with a peak at 25–30 μm, whereas NF68^- ^neurons reached up to 40 μm with a peak at 20–25 μm (Fig. 9B). Triple-immunonegative DiI positive neurons were predominantly small showing a plateau in all size ranges below 30 μm (Fig. 9C). Only 3% (5/155) of the neurons of this neurochemical class were larger than 35 μm in diameter. The Kolmogoroff-Smirnoff-test revealed highly significant differences among ASIC3^+^/TRPV1^-^/NF68^+ ^and ASIC3^+^/TRPV1^-^/NF68^- ^neurons as well as between them and all other neurochemically defined populations. There were no significant differences, however, among ASIC3^-^/TRPV1^+^/NF68^-^, ASIC3^+^/TRPV1^+^/NF68^-^, and triple-negative neurons.

### Comparison between pleural and pulmonary spinal afferents

Triple-negative (ASIC3^-^/TRPV1^-^/NF68^-^) neurons were much more frequent among pulmonary (22%) than among pleural afferents (p < 0.001; Chi^2^-test), whereas neurons with TRPV1-immunoreactivity only (ASIC3^-^/TRPV1^+^/NF68^-^) were more numerous in the neuronal population projecting to the pleura (35% vs. 20%; p < 0.001; Chi^2^-test) (Fig. 4). All other neurochemically defined classes showed no significantly different relative frequencies between pleural and pulmonary afferents. Comparison of neurochemically specified pleural and pulmonary afferents with respect to their size distribution in neither case revealed significant differences (Kolmogoroff-Smirnoff-test).

## Discussion

This study provides the first direct comparison between pleural and pulmonary spinal afferent neurons with respect to the location of their perikarya and neurochemical characteristics, particularly with respect to the expression of acid-sensitive ion channels.

### Origin of pulmonary and pleural afferents

The distribution of retrogradely labelled neurons after tracer injection into the left rat lung observed in the present study with bilaterally located neurons both in vagal sensory ganglia and in DRG with a peak at ipsilateral segmental levels Th1-Th2 generally matches the description given also for the rat by Springall et al. [2] and is similar to that reported for the innervation of the guinea-pig right lung [3]. Application of Fast Blue to murine stem bronchi was followed by labelling of vagal sensory ganglia and DRG neurons with a peak at levels Th3-Th4 [5]. The very few labelled spinal motoneurons that we observed after lung tracing at segmental level C3 might project to the infrahyoid muscles that cover the trachea ventrally [34], since we applied tracer via an open cervical access, and it cannot be excluded that minimal tracer leakage occurred through the slit in the trachea through which the cannula was inserted. Since corresponding segmental DRG levels were excluded for immunohistochemical investigation which was restricted to levels Th1-2, this possible tracer leakage has no influence on the data reported here for spinal pulmonary afferents.

The existence of sensory nerve endings in the parietal pleura has been described earlier [14,15,35,36], but retrograde neuronal tracing studies on their origin have not been reported yet. The occurrence of retrogradely labelled neurons in DRG of both sides after unilateral tracer application to the pleura, as observed in the present study, may appear to be surprising at the first sight, but circular fenestrations in the retrocardiac mediastinal pleura connect the right and left pleural cavities in the rat [37,38] so that tracer is likely to have access to both compartments. These fenestrations are present in the rodent lung where at several sites the pleura of left and right lung are in contact. However their presence in the human lung, where almost no close contacts between the pleura of left and right lung exist, is highly unlikely. Sites of location of retrogradely labelled neurons after tracer application onto the costal pleural were DRG at the segmental level of tracer application, at segmental levels C4-C6, Th1-Th2, and sensory vagal ganglia. The latter (DRG Th1-2, jugular-nodose ganglia) are known to provide a large number of sensory fibres to the lungs as shown earlier [2,3] as well as in our present pulmonary tracing experiments, and tracer application into the pleural cavity is unavoidably accompanied by tracer uptake at the pulmonary pleural surface. Hence, neurons at these locations are likely to represent, at least to a large proportion, pulmonary afferents, and were not investigated immunohistochemically in the present study when the focus was directed onto pleural innervation. Segmental levels C4-C6 release the phrenic nerve who contributes, besides fibres to the serosal surfaces of the diaphragm and mediastinal pleura [14], also a significant number of nerve fibres to the pericardium [39,40]. Since, in the rat, pleural and pericardial cavities are connected by pores [38] and, accordingly, tracer was also observed at the pericardium in our study, we chose not to investigate immunohistochemically neurons of segemental levels C4-C6, because they are likely to represent a mixed population of pleural and pericardial afferents. On the other hand, retrogradely labelled DRG neurons at segmental levels Th3-Th6 shall be dominated by far by pleural afferents, since pericardial tracer injection in the rat predominantly results in labelling of DRG neurons more cranially (C8-Th3) [40-42]. Access of the tracer to underlying intercostal muscle with the consequence of labelling of muscle afferents was excluded by the absence of motoneuron labelling at the corresponding spinal cord segment, while they were readily labelled in the control where tracer was directly injected into the intercostal muscle. Hence, Th3-Th6 DRG neurons were chosen for further immunohistochemical investigation.

### Neurochemical and strucutural characteristics of pleural and pulmonary afferents

Expression of at least one acid-sensitive channel, TRPV1 or ASIC3, is a major characteristic of spinal thoracic pleural afferents, since only 3% of retrogradely labelled neurons contained neither TRPV1- nor ASIC3-immunoreactivity. In this aspect, pleural afferents showed the major difference to pulmonary afferents among the features investigated in the present study, since 22% of neurons retrogradely labelled from the lung were TRPV1- and ASIC3-negative. The percentage of TRPV1-immunoreactive neurons innervating peripheral airways differs between rat and mouse. Thai Dinh et al. [5] found only about 12% TRPV1- immunoreactive DRG neurons after tracing of the mouse left main bronchus. Reasons for this could be species-specific differences or different immunohistochemistry and tracing strategies.

In view of the involvement of the TRPV1- and ASIC3 receptor in perception of painful stimuli [43-45], this finding correlates with the clinically known high sensitivity of the peripheral pleura to nociceptive stimuli.

Studies on human skin using direct infusion of acidic solutions and channel inhibitors indicate that ASICs are responsible for pain perception at a pH ≥ 6.0 while an additional contribution of TRPV1 to pain perception occurs under more severe acidification (pH = 5.0) [24]. ASIC3-immunoreactivity without TRPV1-immunoreactivity was observed in almost half of the pleural afferents (48%) and in quite similar proportion (44%) in pulmonary afferents. These relative proportions might be a slight overestimation of the situation under physiological conditions, since tracer application caused, to some extent, local inflammation, and inflammatory conditions lead to increased ASIC3 transcription in vivo and to an increased number of ASIC3 expressing neurons in vitro [46,47]. Roughly 50% of ASIC3-immunoreactive retrogradely labelled neurons had small diameter and did not contain NF68. Just size alone allows to classify them with rather high likelyhood as perikarya giving rise to C/Aδ-fibres [28,30,31], and lack of NF68-immunoreactivity even more points to a predominance of C-fibres neurons. Three differently-sized neurofilaments (heavy, medium, light = 61–68 kDa) assemble to constitute the most abundant structural component of large myelinated axons [48,49], among which the medium-sized neurofilament is essential for the myelin-directed "outside-in" signalling that mediates axonal radial growth [50]. In rat DRG, all neurons with conduction velocity below 1.3 m/s were not distinctly labelled with an antibody against the heavy neurofilament, and none of the negative neurons conducted faster than 2 m/s [28]. Targeted disruption of the light neurofilament causes extensive axonal loss and reduction of calibre and conduction velocity that is even more severe than in deficiency of medium or heavy neurofilaments (for review, see [49], and double-labelling immunofluoresence studies of rat DRG showed only 6% overlap of immunoreactivity against light neurofilament and peripherin, a maker for unmyelinated neurons [29]. Thus, from the present data it can be inferred that about 25% of rat pleural spinal afferents are slow conducting fibres with sensitivity to minor lowering of pH, and this correlates with an analysis of 41 slowly conducting fibres in the rabbit phrenic nerve with terminal fields in the mediastinal pleura, where 31% were activated by acidified synthetic interstitial fluid, pH 6.1 [14].

Besides its proton sensitivity, an involvement of ASIC3 in the perception of mechanical stimuli has also been proposed [27,51] although an electrophysiological study on cultured DRG neurons from wild-type and ASIC3 null mutant mice failed to detect a contribution of ASIC3 to mechanically activated currents [52]. Of course, both functions are not exclusive to each other, and it is characteristic of nociceptors that they are polymodal, responding to a variety of stimuli including both chemical and mechanical stimuli [45,53]. Indeed, among rabbit parietal pleural afferents, 70% are multimodal receptors [15]. In a rat skin-nerve preparation, continuous infusion of saline of pH 6.1–6.9 increases discharge rate of approximately 40% of polymodal afferent C-fibres and, after repeated exposure to low pH, the mechanical threshold of cutaneous nociceptors is decreased [54]. In mice, repeated intramuscular acid injections cause chronic mechanical hyperalgesia mediated via Aδ-fibres which is dependent on the expression of ASIC3 as shown in ASIC3 knockout mice [25]. It is tempting to assume that similar mechanisms operate in pleural afferents, and that the clinically known painful sensation to otherwise subthreshold mechanical stimuli, such as respiratory movements, in pleuritic conditions may be due to tissue acidification during inflammation with resulting sensitization of ASIC3-carrying afferents.

ASIC3-immunoreactivity in retrogradely labelled TRPV1-negative neurons was not restricted to small, NF68-negative neurons but occurred also in NF68-immunoreactive perikarya with larger soma diameter. In fact, practically all of the retrogradely labelled NF68-positive neurons also exhibited ASIC3-immunoreactivity. The presence of ASIC3 in both medium-sized and larger rat DRG somata has been reported earlier by in-situ hybridization and immunohistochemistry [55,56], and soma size and NF68 content allow to classify them with high likelihood as neurons conducting with A-velocity [55,56]. Indeed, electrophysiological recordings from the murine saphenous nerve showed altered properties of Aδ-fibres in ASIC3 null mutants in that the mechanical threshold of RARs, but not that of SARs, was lowered [27]. As for the parietal pleura, data are available only for the rabbit where all mechanoreceptors exhibit SAR properties, and those with fastest conduction velocity (11.0 ± 1.3 m/s) are purely mechanosensitive [15]. These populations are likely to correspond to the presently identified ASIC3/NF68-immunoreactive DRG neurons projecting to the lung and pleura.

Small-sized NF68-negative neurons with TRPV1-immunoreactivity constituted the largest single population of DRG neurons retrogradely labelled from the pleura (35%), and were also abundant (20%), although significantly less frequent compared to pleural afferents, among those retrogradely labelled from the lung. In case of TRPV1, local inflammation due to tracer injection is not likely to have increased the number of immunolabelled neurons, since peripheral inflammation does increase TRPV1 protein, but most likely via a post-transcriptional mechanism, thereby not affecting the number of neurons expressing it [57-59]. An increase in TRPV1-immunoreactive fibres has recently been described in the airway mucosa of patients with chronic cough [23]. Spinal afferents, however, do not contribute significantly to the cough reflex, since it is effectively blocked by cooling of the vagus nerve [60] but unaffected in subjects with cervical spinal cord injury [61]. Several studies conducted at a variety of systems have identified small TRPV1-positive neurons as C-fibre nociceptors [20,62,63], and it is reasonable to assume a similar function for these pleural and pulmonary DRG afferents. Consistent with this view, 36% of afferents from the rabbit parietal pleura were activated by direct capsaicin application in the study of Jammes et al. [15].

Colocalization of ASIC3- and TRPV1-immunoreactivities was observed in roughly 10% of retrogradely labelled DRG neurons, both among pleural and pulmonary afferents. So far, only few previous reports have indicated their mere occurrence. Whereas electrophysiological recordings of acutely dissociated rat DRG neurons showed no co-occurrence of TPRV1 and ASIC-like currents [64], co-occurrence in 12% of control neurons and in 18% of cells after treatment with proinflammatory mediators have been reported by Mamet et al. [47]. The co-expression of channels that have different pH optima of sensitivity may confer acid sensitivity over a wider pH range, but direct experiments as to the specific function of this subclass of neurons are lacking.

## Conclusion

Spinal afferents to rat lung and pleura express at least two different acid-sensitive channels that make them suitable to monitor tissue acidification. Whereas roughly one fifth of pulmonary spinal afferents contains neither of these channels, at least one is expressed by nearly all (97%) of pleural spinal afferents. Patterns of co-expression of these channels, soma size and neurofilament content allow defining subgroups of neurons that can be inferred to subserve different functions. The significantly higher prevalence of TRPV1^+^/ASIC3^- ^neurons among pleural afferents probably reflects the high sensitivity of the parietal pleura to painful stimuli.

## Authors' contributions

MG, TH, WK, VG and RVH carried out the tracing and the immunohistochemistry. WK and RVH were involved in the design of the study and participated in writing and preparation of the manuscript and in the statistical analysis. The data presented in the manuscript are part of the doctoral thesis of MG and TH.
